# Activating AMPK improves pathological phenotypes due to mtDNA depletion

**DOI:** 10.1111/febs.70006

**Published:** 2025-02-07

**Authors:** Gustavo Carvalho, Tran V. H. Nguyen, Bruno Repolês, Josefin M. E. Forslund, W. M. Ruchitha Rukmal Wijethunga, Farahnaz Ranjbarian, Isabela C. Mendes, Choco Michael Gorospe, Namrata Chaudhari, Micol Falabella, Mara Doimo, Sjoerd Wanrooij, Robert D. S. Pitceathly, Anders Hofer, Paulina H. Wanrooij

**Affiliations:** ^1^ Department of Medical Biochemistry and Biophysics Umeå University Umeå Sweden; ^2^ Department of Neuromuscular Diseases UCL Queen Square Institute of Neurology London UK; ^3^ Clinical Genetics Unit, Department of Women and Children's Health Padua University Padua Italy; ^4^ NHS Highly Specialised Service for Rare Mitochondrial Disorders, Queen Square Centre for Neuromuscular Diseases The National Hospital for Neurology and Neurosurgery London UK

**Keywords:** AMP‐activated protein kinase, AMPK, mitochondrial DNA depletion, polymerase ɣ

## Abstract

AMP‐activated protein kinase (AMPK) is a master regulator of cellular energy homeostasis that also plays a role in preserving mitochondrial function and integrity. Upon a disturbance in the cellular energy state that increases AMP levels, AMPK activity promotes a switch from anabolic to catabolic metabolism to restore energy homeostasis. However, the level of severity of mitochondrial dysfunction required to trigger AMPK activation is currently unclear, as is whether stimulation of AMPK using specific agonists can improve the cellular phenotype following mitochondrial dysfunction. Using a cellular model of mitochondrial disease characterized by progressive mitochondrial DNA (mtDNA) depletion and deteriorating mitochondrial metabolism, we show that mitochondria‐associated AMPK becomes activated early in the course of the advancing mitochondrial dysfunction, before any quantifiable decrease in the ATP/(AMP + ADP) ratio or respiratory chain activity. Moreover, stimulation of AMPK activity using the specific small‐molecule agonist A‐769662 alleviated the mitochondrial phenotypes caused by the mtDNA depletion and restored normal mitochondrial membrane potential. Notably, the agonist treatment was able to partially restore mtDNA levels in cells with severe mtDNA depletion, while it had no impact on mtDNA levels of control cells. The beneficial impact of the agonist on mitochondrial membrane potential was also observed in cells from patients suffering from mtDNA depletion. These findings improve our understanding of the effects of specific small‐molecule activators of AMPK on mitochondrial and cellular function and suggest a potential application for these compounds in disease states involving mtDNA depletion.

## Graphical abstract

In a cellular model of mitochondrial disease characterized by progressive mitochondrial DNA (mtDNA) depletion, we found that advancing mitochondrial dysfunction triggers the early and specific activation of the mitochondria‐associated pool of AMP‐activated protein kinase (AMPK), a master regulator of energy homeostasis. Pharmacological stimulation of AMPK activity by an AMPK‐specific small‐molecule agonist had beneficial effects, suggesting a potential utility for such compounds in disease states involving mtDNA depletion.

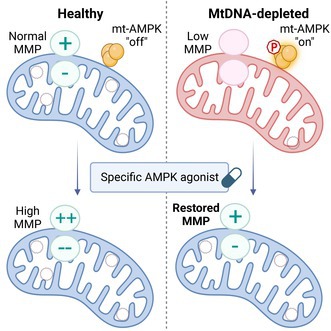

**Abbreviations**
ACC: acetyl‐coenzyme A carboxylase
ADaM: allosteric drug and metabolite‐binding site
AICAR: 5‐aminoimidazole‐4‐carboxamide ribonucleoside
AMPK: AMP‐activated protein kinase
C: complex (of the respiratory chain): I, II, III, IV, and V
CBS: cystathionine‐β‐synthase
Ctrl: control
DOI: days of induction
MDS: mtDNA depletion syndrome
MMP: mitochondrial membrane potential
mtDNA: mitochondrial DNA
NAO: 10‐N‐nonyl acridine orange
OXPHOS: oxidative phosphorylation
PGC‐1⍺: peroxisome proliferator‐activated receptor ɣ coactivator 1⍺
Polɣ: mtDNA polymerase ɣ
TFAM: mitochondrial transcription factor A
TMRE: tetramethylrhodamine ethyl ester
WB: western blot

## Introduction

Mitochondrial disorders are a heterogeneous group of often debilitating diseases, many of which manifest during childhood. They are caused by pathogenic variants in nuclear or mitochondrial genes that impair respiratory chain function and/or mitochondrial ATP production either directly or indirectly. The first group includes genetic variants in genes directly involved in oxidative phosphorylation (OXPHOS), while the second comprises genes whose gene products are essential for the maintenance or expression of mitochondrial DNA (mtDNA). With a few exceptions, there is currently no cure for mitochondrial diseases, and therapeutic options are thus generally limited to management of the patients' individual symptoms [1, 2]. Continued research into potential treatments and their mechanisms of action is thus called for.

An experimental approach that has shown promise in animal models of mitochondrial disease is the general induction of mitochondrial biogenesis, achieved for example by activating the key energy sensor and metabolic regulator, the AMP‐activated protein kinase (AMPK) [3, 4]. AMPK becomes activated upon energetic stresses manifesting as a decreased ratio of ATP to AMP and/or ADP, and phosphorylates its targets to shift metabolism from anabolism to catabolism [5]. This energy‐metabolic reprogramming involves upregulation of lipid and glucose breakdown, increased autophagy and mitophagy, and a boost in lysosomal and mitochondrial biogenesis [6]. More chronic activation of AMPK induces the expression of a core group of genes including peroxisome proliferator‐activated receptor ɣ coactivator 1 ⍺ (PGC‐1⍺), a positive regulator of mitochondrial gene expression from the nuclear genome [7, 8, 9]. In addition to transcriptional stimulation, AMPK is thought to stimulate PGC‐1⍺ activity through a combination of direct or indirect posttranslational modifications [6], consequently promoting the expression of mitochondrial transcription factor A (TFAM), the high‐mobility group protein that is required for the maintenance and expression of mtDNA [10, 11, 12]. Increased TFAM levels lead to elevated mtDNA copy number, allowing for the overall expansion of functional mitochondria [13, 14]. Thus, activation of the AMPK‐PGC‐1⍺ axis increases mitochondrial biogenesis to augment respiratory capacity in response to a low cellular energy state.

In line with this concept, the pharmacological activation of AMPK using the agonist 5‐aminoimidazole‐4‐carboxamide ribonucleoside (AICAR) has been shown to improve muscle function in different models of mitochondrial myopathy caused by complex IV (CIV) defects [3, 4]. However, neither of the above‐cited studies found evidence for the expected increase in mitochondrial biogenesis in terms of elevated mtDNA copy number or mitochondrial mass, leaving the mechanisms underlying these positive effects of AICAR somewhat unclear [3, 4]. *In vivo*, AICAR is converted to the inosine pathway intermediate ZMP that acts as an AMP analog. In addition to binding and stimulating AMPK, ZMP modulates the activity of other AMP‐regulated enzymes such as glycogen phosphorylase and fructose‐1,6‐bisphosphatase [15, 16, 17]. Therefore, some of the positive effects of AICAR in models of mitochondrial disease might potentially be attributable to AMPK‐independent actions of the drug.

More specific, non‐AMP‐mimetic AMPK agonists such as A‐769662 and compound 991 have been developed [18, 19, 20], but have not yet been as widely adopted as AICAR. Therefore, their impact on mitochondrial function and especially mtDNA levels has not been conclusively determined. However, A‐769662 and other specific AMPK agonists showed positive effects in a panel of patient‐derived cell lines with genetically heterogeneous forms of mitochondrial defects [21]. The group of cell lines benefiting from AMPK agonists included not only cell lines with direct defects in the OXPHOS machinery, but also ones manifesting mitochondrial dysfunction due to defects in mtDNA maintenance. Moreover, a recent study reported an increase in both mitochondrial mass and mtDNA copy number in HEK293 cells treated with compound 991 [7]. These findings raise the possibility that specific AMPK agonists may be beneficial even in cases with indirect OXPHOS defects resulting from decreased mtDNA copy number, *that is*, mtDNA depletion.

MtDNA depletion syndromes (MDS) are associated with severe, infantile‐onset mitochondrial dysfunction with highly tissue‐specific manifestations typically caused by pathogenic variants of enzymes involved in nucleotide metabolism or mtDNA replication, such as the mtDNA polymerase ɣ (Polɣ) [22, 23, 24]. The Polɣ holoenzyme consists of one catalytic PolɣA subunit that associates with a homodimer of accessory PolɣB subunits which improve processivity of the holoenzyme [25, 26, 27, 28]. The catalytic activity of PolɣA relies on the evolutionarily conserved aspartate residues D890 and D1135 [29]. Accordingly, inducible overexpression of the PolɣA^D890N^ or PolɣA^D1135A^ variants in cultured human cells containing an endogenous wild‐type copy of the *POLG* gene exerts a dominant‐negative phenotype with prominent mtDNA replication stalling and subsequent rapid mtDNA depletion, acting as an excellent cell model for MDS progression [30, 31].

In this study, we use a Flp‐In T‐REx 293 cell line with inducible expression of PolɣA^D1135A^ to address the role of AMPK activity during escalating mitochondrial dysfunction caused by severe mtDNA depletion. We first delineate the timing of events during the advancing decline in mtDNA levels caused by the expression of the dominant‐negative D1135A variant. We demonstrate that AMPK activation is an early event that occurs before the cellular energy state or mitochondrial membrane potential is measurably affected, confirming that AMPK successfully restores energy homeostasis during the early stages of mitochondrial dysfunction. Notably, the observed activation preferentially affects AMPK molecules in the mitochondrial fraction of the cell, while cytosolic AMPK is not activated. Next, we show that AMPK contributes to the maintenance of mitochondrial membrane potential even under basal conditions and that stimulation of AMPK activity using the specific agonist A‐769662 is sufficient to fully restore the mitochondrial membrane potential in mtDNA‐depleted cells. This positive impact of A‐769662 in mtDNA‐depleted cells was accompanied by a small increase in the levels of mtDNA and respiratory chain subunits. Treatment with the AMPK agonist also elevated mitochondrial membrane potential in cell lines derived from patients suffering from Polɣ‐associated mitochondrial myopathy, but then through an mtDNA‐independent mechanism. Our findings thus uncover differential effects of the AMPK agonist in control cells and ones suffering from severe mtDNA depletion.

## Results

### The progressive mtDNA depletion induced by Polɣ^D1135A^ expression impairs OXPHOS and cell proliferation within 4–6 days

**Fig. 1:**
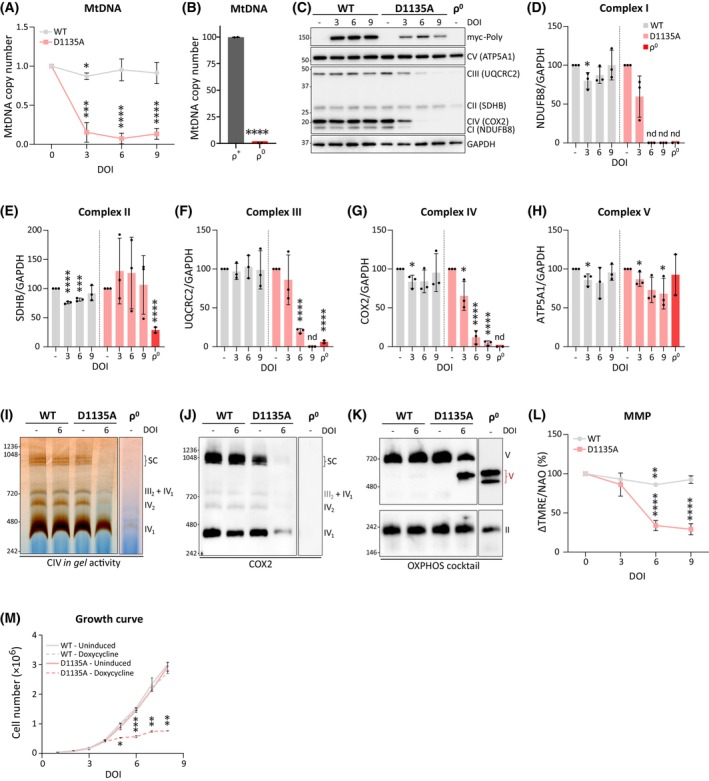
Progressive loss of mitochondrial function in a model of mtDNA depletion. (A) Relative mtDNA copy number following the overexpression of Polɣ^wt^ (*gray*) and the dominant‐negative Polɣ^D1135A^ (*pink*) over the course of 3, 6, and 9 days of induction (DOI) with 3 ng·mL^−1^ doxycycline. The relative mtDNA copy number was normalized to the respective uninduced cells (0 DOI); *n* = 3. (B) MtDNA copy number is undetectable in HEK293 T‐REx ρ^0^ cells compared to ρ^+^ cells; *n* = 2. See Materials and Methods for details on ρ^0^ cell generation. (C) Expression of subunits of the mitochondrial respiratory complexes and the ATP synthase (complexes I to V) after 3, 6, and 9 days of induction of Polɣ^wt^ and Polɣ^D1135A^ (indicated as myc‐Polɣ) and HEK293 ρ^0^ cells were assessed by WB using an OXPHOS cocktail antibody. The position of protein molecular weight markers (kDa) is indicated on the left and protein names (and their respective respiratory complexes) are indicated on the right. A representative image of *n* = 3. The same cellular lysates were used for the experiment in Fig. 2D. (D–H) Quantification of the blots shown in (C), with Polɣ^wt^ (*gray*), Polɣ^D1135A^ (*pink*), and ρ^0^ cells (*red*). Protein levels were normalized against GAPDH. (I) The function of CIV and supercomplexes containing CIV was assessed by *in‐gel* activity assay after BN‐PAGE from the indicated cell lines. Representative image of *n* = 3. (J) Immunoblotting with COX2 antibody to assess the integrity of CIV following BN‐PAGE. Representative image of *n* = 3. (K) Immunoblotting with OXPHOS cocktail antibody to detect CI, CII, and CV in a BN‐PAGE gel. The following complexes and supercomplexes are indicated: (IV_1_) CIV monomer; (IV_2_) CIV dimer; (III_2_ + IV_1_) CIII dimer and CIV monomer; (SC) respiratory supercomplexes formed by CI, CIII, and CIV; (V) complex V. In cells expressing Polγ^D1135A^ for 6 days and in ρ^0^ cells, the accumulation of partially assembled CV (in red) is detected. Representative image of *n* = 3. (L) Mitochondrial membrane potential (MMP) was estimated by flow cytometry using TMRE dye. Nonmitochondrial TMRE fluorescence was subtracted from cells treated with the mitochondrial uncoupler BAM15 (ΔTMRE). TMRE signal was adjusted to mitochondrial mass estimated by staining mitochondria with 10‐N‐nonyl acridine orange (NAO). Relative MMP is expressed as ΔTMRE/NAO; *n* = 3. (M) Growth curve of uninduced cells or induced cells overexpressing either Polɣ^wt^ or Polɣ^D1135A^ (*doxycycline*) was obtained by counting the absolute number of cells over the course of 8 days; *n* = 2. Results were analyzed by unpaired *t* test; error bars in the graphs represent standard deviation (*n*, number of biological replicates; nd, not determined; DOI, days of induction; *P ≤ 0.05; **P ≤ 0.01; ***P ≤ 0.001; ****P ≤ 0.0001).

We used the previously established Flp‐In T‐REx 293 cell line to express the dominant‐negative D1135A variant of the mitochondrial DNA polymerase PolɣA (henceforth referred to as Polɣ^D1135A^) in a doxycycline‐inducible manner [30]. A similar cell line expressing the wild‐type PolɣA (Polɣ^wt^) was used as a control. To better mimic physiological energy metabolism and mitochondrial function, the cells were maintained in low‐glucose media (5 mm glucose). The expression of Polɣ^wt^ and Polɣ^D1135A^ was induced by the addition of 3 ng·mL^−1^ of doxycycline, a low concentration that was selected because it decreased mtDNA levels to below 15% of starting levels by day 3 in Polɣ^D1135A^‐expressing cells (Fig. 1A; ρ^0^ cells entirely lacking mtDNA were generated for use as a control, Fig. 1B). No leaky expression of Polɣ^wt^ or Polɣ^D1135A^ was observed in the absence of doxycycline (Fig. 1C).

To follow the dynamics of the mitochondrial and cellular effects of mtDNA depletion, the expression of Polɣ^D1135A^ was induced, and samples were collected 3, 6, and 9 days after induction. Cells expressing Polɣ^D1135A^ exhibited a considerably decreased copy number of 8–15% of starting levels across all three postinduction timepoints (Fig. 1A), while the protein levels of respiratory chain (RC) subunits showed a decreasing trend over the time course (Fig. 1C–H). Subunits of complex IV and V were mildly affected already on day 3, and by day 6, all complexes except for the fully nuclear‐encoded CII showed diminished protein levels. In line with the trend in the Flp‐In T‐REx 293 ρ^0^ control cells, subunits of complexes I, III, and IV were most severely affected, while the decrease in ATP5 (CV) levels was more moderate (ca 70% of initial protein levels remaining on day 9). In contrast to Polɣ^D1135A^, the expression of Polɣ^wt^ had little or no impact on copy number or RC complex levels, confirming that the observed effects were not related to an overwhelmed protein import system upon overexpression of a mitochondrially targeted protein. Blue native analysis confirmed a pronounced loss of assembled RC complexes and supercomplexes in Polɣ^D1135A^ cells on day 6 of induction, including the appearance of a smaller subcomplex of CV that was also observed in ρ^0^ cells by us and others (Fig. 1I–K) [32]. In line with the severe decrease in CIV protein levels, the activity of the complex on day 6 of induction was notably diminished compared to uninduced Polɣ^D1135A^ cells (Fig. 1I–K). Accordingly, the mitochondrial membrane potential (MMP), measured using the cationic dye tetramethylrhodamine methyl ester perchlorate (TMRE) and normalized to mitochondrial mass assessed by 10‐N‐nonyl acridine orange (NAO), diminished to 35% of starting levels by day 6 (Fig. 1L). Cell proliferation stagnated already on day 5, suggesting that the decrease in OXPHOS and MMP observed in day 6 samples may be physiologically relevant already on day 5 when no samples were collected for protein or MMP analysis (Fig. 1M).

### Mitochondria‐associated AMPK is activated early on during the course of the mitochondrial dysfunction

**Fig. 2:**
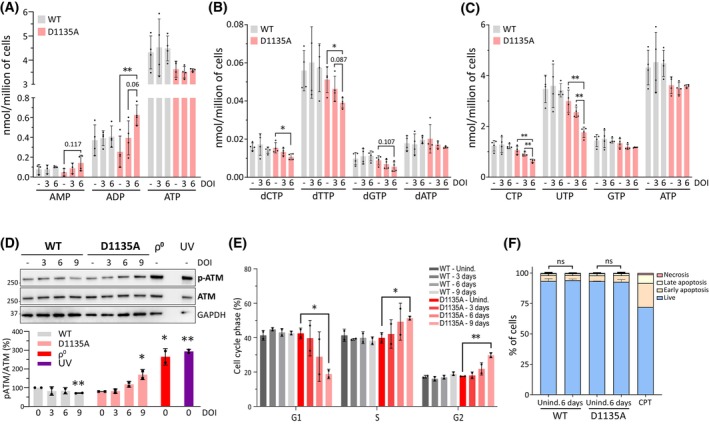
MtDNA loss leads to energy imbalance, decreased pyrimidine nucleotide pools, and eventual checkpoint activation. (A) Levels of AMP, ADP, and ATP in cells either uninduced (day 0) or expressing Polɣ^wt^ (*gray*) or Polɣ^D1135A^ (*pink*) for 3 and 6 days; *n* = 4. (B, C) Levels of dNTPs and NTPs are shown for uninduced cells (*0 d*) and cells expressing Polɣ^wt^ (*gray*) or Polɣ^D1135A^ (*pink*) for 3 or 6 days; *n* = 4. (D) Immunoblotting shows levels of phosphorylated ATM (p‐ATM) and total ATM in cells expressing Polɣ^WT^ and Polɣ^D1135A^ for 3, 6, and 9 days, and in ρ^0^ cells as well as in control cells treated with UV‐light. Quantification of ATM phosphorylation is shown in the lower panel; *n* = 2. The same cellular lysates were used for the experiment in Fig. 1C. (E) Cell cycle phases (G1, S and G2) were assessed by flow cytometry in cells expressing Polɣ^wt^ and Polɣ^D1135A^ for 3, 6, and 9 days; *n* = 2. (F) Cell death was measured in cells expressing Polɣ^wt^ and Polɣ^D1135A^ for 6 days; *n* = 2. Treatment with camptothecin (CPT), a topoisomerase inhibitor that induces DNA damage, was included as a positive control for cell death once because it is a technical control only, *n* = 1. Results were analyzed by unpaired *t* test; error bars in the graphs represent standard deviation. (*n*, number of biological replicates; *P ≤ 0.05; **P ≤ 0.01).

To further assess the effects of mtDNA depletion on energy metabolism, we next used HPLC to quantify the levels of adenine nucleotides in Polɣ^D1135A^ or Polɣ^wt^‐expressing cells before induction, as well as 3 or 6 days after the addition of doxycycline. In line with the observed MMP defect, the ADP level in Polɣ^D1135A^‐expressing cells was elevated at day 6, and AMP levels showed a reproducible increase that however failed to reach statistical significance (*P* = 0.117; Fig. 2A). The cells' energy status, appraised by the ratio of ATP to the sum of AMP and ADP, thus decreased 2.5‐fold compared to uninduced Polɣ^D1135A^ cells (with a computed ATP/(AMP + ADP) ratio of 12.0 and 4.7 in uninduced and day‐6 cells, respectively). Further analysis of nucleotide levels revealed a decline in all pyrimidine nucleoside triphosphates (CTP, UTP, dCTP, and dTTP) in Polɣ^D1135A^ cells, which is an expected consequence of the respiratory chain deficiency impairing pyrimidine synthesis (Fig. 2B,C) [33, 34]. The Polɣ^D1135A^ cells also exhibited activation of the ATM checkpoint kinase and an altered cell cycle profile (Fig. 2D,E). No signs of increased apoptosis were detected (Fig. 2F). These findings are in good agreement with the previously reported cell cycle alterations and ATM activation observed in response to mtDNA instability, and the fact that mitochondrial dysfunction can lead to nuclear DNA instability through a host of different mechanisms [35, 36, 37, 38].

**Fig. 3:**
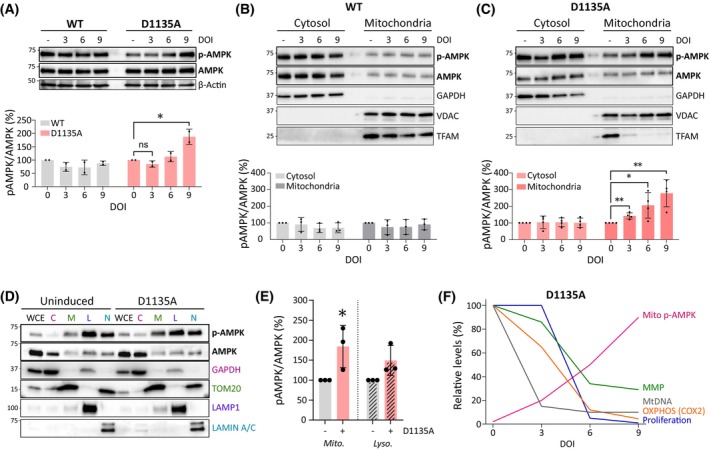
MtDNA loss leads to the preferential activation of mitochondria‐associated AMPK. (A) Immunoblotting indicates levels of phosphorylated AMPK (p‐AMPK) and total AMPK in cells overexpressing Polɣ^wt^ and Polɣ^D1135A^. β‐Actin was used as loading control. A representative image of *n* = 2 is shown. (B, C) Cell fractionation was performed to show the subcellular localization of AMPK. Immunoblotting shows the distribution of p‐AMPK and AMPK in the cytosolic and mitochondrial fraction of cells overexpressing Polɣ^wt^ (*n* = 3) and Polɣ^D1135A^ (*n* = 4). Quantification of the p‐AMPK/AMPK ratios is shown in the bottom panel. GAPDH and VDAC are shown as a purity control for the cytosolic and mitochondrial fractions, respectively. TFAM levels provide an indirect estimation of mtDNA copy number (see also Fig. 1A). (D) Density gradient centrifugation was used to separate the crude mitochondrial fraction of uninduced or 4‐days induced Polɣ^D1135A^ cells into mitochondrial (*M*) and lysosomal (*L*) fractions; the whole‐cell extract (*WCE*), and cytosolic (*C*) and nuclear (*N*) fractions are shown for reference only. GAPDH, TOM20, LAMP1, and Lamin A/C are shown as purity controls for the cytosolic, mitochondrial, lysosomal, and nuclear fractions, respectively. Location of molecular weight marker bands is indicated on the left (kDa). (E) Quantification of the p‐AMPK/AMPK ratios in the pure mitochondrial and lysosomal subfractions from D. The intensity of the p‐AMPK and AMPK bands in the uninduced sample of each respective subfraction was set to 1. The *P*‐value for the comparison of the p‐AMPK/AMPK ratio in uninduced *vs*. induced mitochondrial fractions is 0.0515. (F) A schematic summarizing the advance of the cellular phenotypes associated with the progressive loss of mtDNA shown in Figs 1, 2, 3. Results were analyzed by unpaired *t* test; error bars in the graphs represent standard deviation. (*n*, number of biological replicates; ns, not significant; DOI, days of induction; *P ≤ 0.05; **P ≤ 0.01).

Given the altered ATP/(AMP + ADP) ratio in Polɣ^D1135A^‐expressing cells, we next examined the status of the AMPK energy sensor that is found in many different subcellular localizations including the cytosol, nucleus, and associated with the lysosomes or the outer mitochondrial membrane [39, 40]. Specifically, we were interested in understanding when over the course of the progressive mitochondrial dysfunction in the Polɣ^D1135A^ cells AMPK becomes activated and thus phosphorylated. Analysis of AMPK phosphorylation on threonine‐172 in whole‐cell lysates suggested that AMPK was not activated until day 9 of induction (Fig. 3A). However, evaluation of AMPK in different subcellular localizations revealed that mitochondria‐associated AMPK underwent activatory phosphorylation already on day 3, while the phosphorylation state of cytosolic AMPK remained at basal levels throughout the time course (Fig. 3B,C). Notably, the energy stress in Polɣ^D1135A^ cells did not alter the partitioning of AMPK between the cytosolic and the crude mitochondrial fraction, indicating no significant recruitment of additional AMPK complexes to the mitochondrial surface (Fig. 3C). Because the crude mitochondrial fractions shown in Fig. 3B,C contain other organelles including lysosomes that AMPK also associates with [39], we next used density gradient centrifugation to separate the crude mitochondrial fraction into pure mitochondrial and lysosomal subfractions (Fig. 3D). Western blot analysis of the pure mitochondrial subfraction confirmed that mitochondria‐associated AMPK was activated following the expression of Polɣ^D1135A^ (Fig. 3D,E). As expected, the induced expression of Polɣ^wt^ did not result in activation of AMPK in any cellular compartment (Fig. 3B). These results show that the energy defect caused by mtDNA depletion in Polɣ^D1135A^ cells primarily activates AMPK that is already associated with mitochondria.

The dynamics of the ensuing mitochondrial dysfunction in our experimental time course are summarized in Fig. 3F: The first effects on RC protein levels are observed on day 3 of induction, when the activation of mitochondria‐associated AMPK also becomes apparent. Cell proliferation declines by day 5, and by day 6, the cells exhibit full‐blown mitochondrial dysfunction involving diminished levels of most RC proteins, decreased MMP, and an overall decline in cellular energy state.

### AMPK helps maintain basal MMP, and stimulation of AMPK activity rescues the MMP loss in Polɣ^D1135A^ cells

**Fig. 4:**
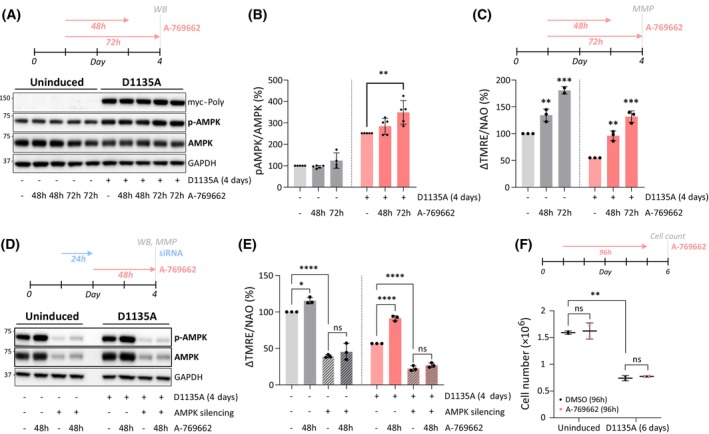
Pharmacological activation of AMPK restores MMP in mtDNA‐depleted cells. (A) Immunoblotting indicates levels of phosphorylated AMPK (p‐AMPK) and total AMPK in uninduced cells and in cells expressing Polɣ^D1135A^ (4‐days induction) prior (−) and following AMPK activation with 100 μm A‐769662 for 48 or 72 h. A representative image of *n* = 4–5 is shown. GAPDH is provided as loading control. (B) Quantification of p‐AMPK/AMPK ratios from A. *n* = 4–5. (C) MMP in uninduced (*gray*) cells and cells expressing Polɣ^D1135A^ (*pink*; 4‐days induction) prior (−) and following AMPK activation with 100 μm A‐769662 for 48 or 72 h; *n* = 3. (D) Immunoblotting indicates levels of phosphorylated AMPK (p‐AMPK) and total AMPK in uninduced cells and in ones expressing PolG^D1135A^ following silencing of AMPK using siRNA against α1/α2 AMPK, or a scrambled siRNA as negative control. A representative image of *n* = 3 is shown. (E) MMP in uninduced (*gray*) cells and cells expressing Polɣ^D1135A^ (*pink*; 4‐days induction) following AMPK silencing (*striped bars*) and/or A‐769662 treatment (*darker shade*) from the experiment in D; *n* = 3. (F) Cell proliferation assessed by cell number after a 96‐h treatment with 100 μm A‐769662 (*pink*) in uninduced cells or ones induced to express Polɣ^D1135A^ for 6 days; *n* = 2. Results were analyzed by unpaired *t* test; error bars in the graphs represent standard deviation. (*n*, number of biological replicates; ns, not significant; *P ≤ 0.05; **P ≤ 0.01; ***P ≤ 0.001; ****P ≤ 0.0001).

Based on the timeline in Fig. 3F, we focused our efforts on Polɣ^D1135A^ cells on day 4 of induction, reasoning that this was a timepoint when the cells were suffering from mild mitochondrial dysfunction that could estimate the situation in mild to moderate mitochondrial disease. We asked whether pharmacological modulation of AMPK activity could alleviate some elements of the mitochondrial dysfunction and treated uninduced or induced Polɣ^D1135A^ cells with the specific AMPK activator A‐769662 for 48 or 72 h, as indicated at the top of Fig. 4A. Treatment with A‐769662 resulted in the expected activation of AMPK as evidenced by the phosphorylation status of AMPK (Fig. 4A,B). Notably, A‐769662 treatment increased mitochondrial membrane potential in a time‐dependent manner, with 72‐h treatment increasing MMP to up to 140% of starting levels in Polɣ^D1135A^‐expressing cells (Fig. 4C). The impact of A‐769662 on MMP was more pronounced in cells with a low starting MMP (80% and 140% increase in uninduced and induced Polɣ^D1135A^ cells after 72 h, respectively) and was sufficient to restore the MMP of induced Polɣ^D1135A^ cells to levels corresponding to the uninduced cells already after 48 h of treatment. We conclude that A‐769662‐mediated AMPK activation is sufficient to correct a considerable drop in mitochondrial membrane potential and that the sustained effect of the activator can be observed at least 24 h after removal of the drug (Fig. 4C; 48‐h treatment).

In contrast to AMP‐mimetic AMPK activators such as AICAR, A‐769662 has been reported to be specific to AMPK [19, 20]. To confirm that the effect of A‐769662 on MMP was AMPK‐dependent, we next silenced the catalytic ⍺‐subunit of AMPK by siRNA treatment and followed the impact of A‐769662 on MMP in uninduced or induced Polɣ^D1135A^ cells. The siRNA treatment resulted in efficient knockdown of AMPK⍺ as well as a clear drop in MMP in both uninduced and induced cells, indicating that AMPK is required for maintaining normal mitochondrial membrane potential (Fig. 4D,E). This finding is in good agreement with the reported impairment of mitochondrial respiration in mouse models lacking ⍺1 and/or ⍺2 subunits of AMPK [41, 42]. Further, as was seen in Fig. 4C, treatment with A‐769662 increased the MMP in both the uninduced and induced cells, and the increase was more substantial in the induced cells that started from a lower MMP (Fig. 4E). However, A‐769662 had no impact on the MMP in cells transfected with an siRNA against AMPK, confirming that the stimulatory effect of A‐769662 on MMP is dependent on the presence of AMPK.

We have previously shown that the cell proliferation defect of *Saccharomyces cerevisiae* cells suffering from mitochondrial dysfunction can be rescued by increasing their MMP [43], and therefore, now analyzed cell proliferation following A‐769662 treatment in uninduced cells and ones induced for 6 days. As shown in Fig. 4F, the rescue in MMP observed after A‐769662 treatment of induced Polɣ^D1135A^ cells was insufficient to improve cell proliferation, indicating that their growth defect in mtDNA‐depleted mammalian cells was due to other consequences of the mitochondrial dysfunction than solely MMP loss.

### The A‐769662‐mediated activation of AMPK improves mtDNA copy number and respiratory chain protein levels in Polɣ^D1135A^ ‐expressing cells but not in control cells

**Fig. 5:**
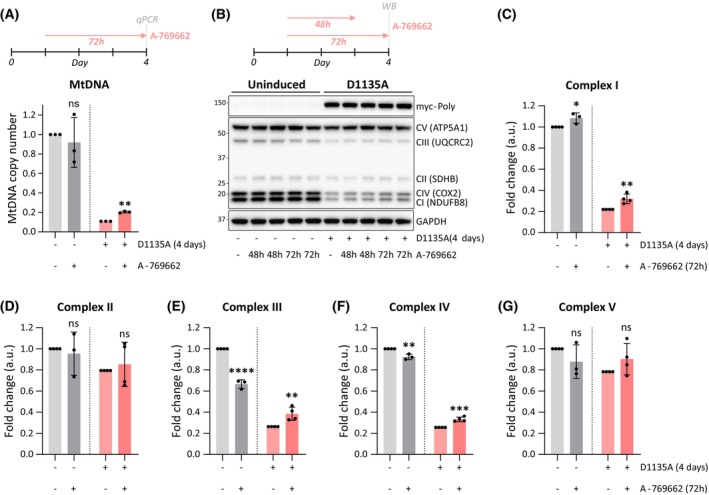
A‐769662 treatment increases mtDNA copy number and RC proteins in Polɣ^D1135A^ cells. (A) Relative mtDNA copy number after 72‐h treatment with A‐769662 (100 μm) reveals increased mtDNA levels in cells expressing Polɣ^D1135A^ (*pink*) for 4 days compared to its respective DMSO control. *n* = 3. (B) Expression of subunits of the mitochondrial respiratory complexes (CI‐CV) after 48‐h and 72‐h treatment with A‐769662 (100 μm) in uninduced cells and in cells expressing Polɣ^D1135A^ (myc‐Polɣ) for 4 days. Protein levels were assessed by WB using an OXPHOS cocktail antibody. Protein molecular weight (kDa) is indicated on the left and protein names (and their respective respiratory complexes) are indicated on the right. A representative image of *n* = 3–4 is shown. The GAPDH and MYC panels of this experiment are also shown in Fig. 4A. (C–G) Quantification of the blots shown in (B); protein levels were normalized against GAPDH. Results were analyzed by unpaired *t* test; error bars in the graphs represent standard deviation. (*n*, number of biological replicates; ns, not significant; *P ≤ 0.05; **P ≤ 0.01; ***P ≤ 0.001; ****P ≤ 0.0001).

Given the positive impact of A‐769962 on the mitochondrial membrane potential of induced Polɣ^D1135A^ cells, we sought to understand the molecular basis of this outcome by exploring the effect of A‐769662 on the levels of mtDNA and RC subunits. A 72‐h treatment of cells with A‐769662 caused a small but statistically significant increase in the mtDNA copy number of induced Polɣ^D1135A^ cells, increasing mtDNA from 11% to 20% of the levels observed in uninduced cells (Fig. 5A). Interestingly, A‐769662 treatment did not alter mtDNA levels in uninduced cells. Analysis of RC protein levels in induced cells revealed a positive effect of A‐769662 on the analyzed CI, CIII, and CIV subunits, but no alteration in CII or CV subunits that were only mildly depleted relative to uninduced cells to begin with (Fig. 5B–G). As was observed with mtDNA copy number, activator treatment had a differential effect on induced and uninduced cells: The protein levels of the latter only increased in one case (CI), while they decreased in two cases (CIII and CIV) and were not affected for CII and CV. Taken together, these results suggest that specific AMPK stimulation can alleviate the effects of mtDNA depletion in Polɣ^D1135A^ cells. However, given that the increase in mtDNA copy number was not observed in uninduced cells that nonetheless exhibited heightened MMP following A‐769662 treatment, we concluded that the mtDNA increase was not the main mechanism through which A‐769662 boosts MMP in uninduced cells.

### AMPK activation by A‐769662 ameliorates ISR and leads to perinuclear mitochondrial clustering

**Fig. 6:**
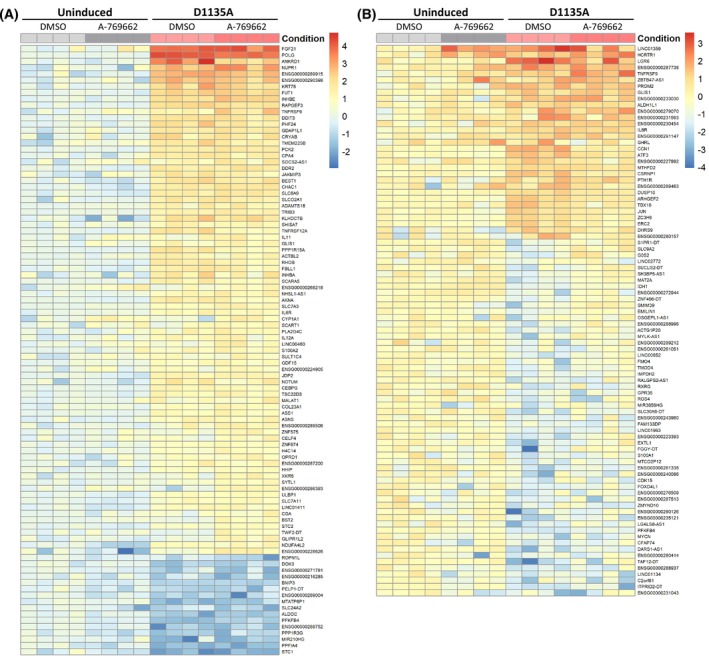
The integrated stress response (ISR) signature of Polɣ^D1135A^ cells is partially blunted following pharmacological activation of A‐769662. (A) RNA‐seq analysis of uninduced or induced (4 d) Polɣ^D1135A^ cells untreated or treated with 100 μm A‐769662 for 72 h was used to identify genes whose expression was altered in at least one of the three conditions when compared to uninduced Polɣ^D1135A^ cells (Table S1). The genes with a log_2_ fold change of ≥1‐fold compared to uninduced Polɣ^D1135A^ cells are shown in the heatmap (Table S2). (B) Heatmap of the 88 ‘rescued’ genes that were differentially expressed in induced Polɣ^D1135A^ cells but not in Polɣ^D1135A^ cells treated with A‐769662 (Table S3). Coloring in the heatmaps is by log_2_FC expression values relative to uninduced and untreated cells.

We next compared the transcriptional profiles of untreated or A‐769662‐treated uninduced or Polɣ^D1135A^‐expressing cells using RNA‐seq analysis. The expression patterns of genes whose expression was at least twofold altered compared to uninduced cells in any of the tested conditions are presented in Fig. 6A (see Tables S1 and S2 for average changes in gene expression values, unfiltered or filtered by log_2_ fold change, respectively). Notably, treatment of uninduced cells with A‐769662 did not lead to any significant gene expression changes (0 differentially expressed genes between uninduced *vs*. uninduced + A‐769662; Table S2), indicating that the impact of the activator on MMP was not achieved through major transcriptional changes. Interestingly, comparison of Polɣ^D1135A^‐expressing cells to uninduced cells identified upregulation of several components of the integrated stress response (ISR), a transcriptional program often observed in cells suffering from various stresses including mitochondrial dysfunction [44, 45]. Upregulated factors in Polɣ^D1135A^ cells included several direct targets of the central ISR transcription factor ATF4 (activating transcription factor 4), such as the C/EBP‐homologous protein (CHOP/DDIT3), the cyclic AMP‐dependent transcription factor ATF3, as well as the GADD34 (gene name *PPP1R15a*) regulatory subunit of the protein phosphatase 1 complex that helps terminate ISR (Fig. 6A; Table S1). Also, the expression of the circulating cytokines fibroblast growth factor 21 (FGF21) and growth/differentiation factor 15 (GDF15), found upregulated in various mitochondrial disease models [46, 47], was significantly induced in Polɣ^D1135A^ cells (Fig. 6A; Table S1). Treatment of Polɣ^D1135A^ cells with A‐769662 restored the expression levels of 88 genes to the normal range; these so‐called “rescued” genes included ATF3 and the ATF4‐binding partner JUN, as well as ALDH1L1 and MTHFD2 of the ISR‐inducible one‐carbon metabolism pathway (Fig. 6B; Table S3). However, other ISR genes such as CHOP/DDIT3 and PPP1R15A remained differentially expressed in Polɣ^D1135A^ cells treated with A‐769662, indicating that the increased MMP caused by the AMPK activator was not able to fully eliminate mitochondrial ISR in these cells (Table S2).

**Fig. 7:**
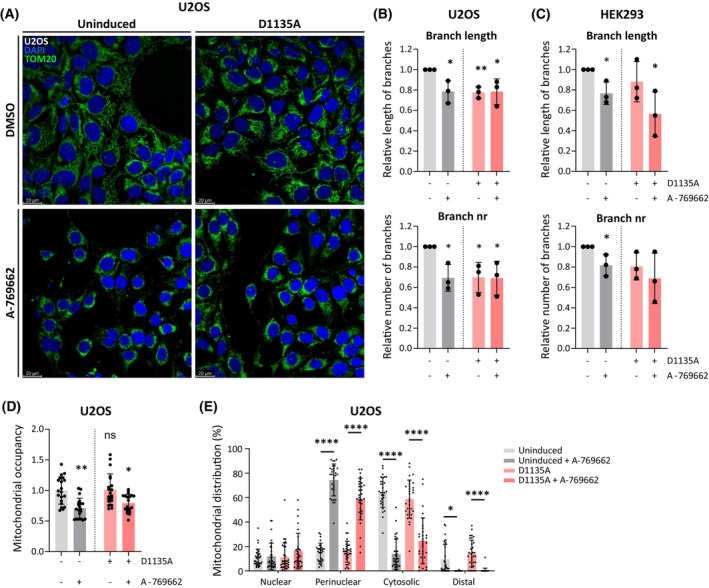
A‐769662‐treated mitochondria are fragmented and accumulate in the perinuclear region. (A) Representative confocal microscopy images from uninduced and induced (6 days) U2OS Polɣ^D1135A^ cells either untreated or treated with 100 μm A‐769662 for 48 h. DAPI‐stained nuclei (*blue*) and mitochondria marked by TOM20 (*green*) are shown; scale bar 20 μm. (B, C) Quantification of features of the mitochondrial network: the mean length of summed branches (*top panel*) and the mean number of network branches (*bottom panel*) in U2OS (B) and HEK293 (C) Polɣ^D1135A^ cells. *n* = 3. (D) Mitochondrial occupancy was measured as the fraction of U2OS Polɣ^D1135A^ cell area occupied by mitochondrial (TOM20) signal in the experiment shown in A. The average of the untreated and uninduced cells was set to 1; *n* = 20. (E) The subcellular distribution of mitochondria in the U2OS Polɣ^D1135A^ cells from the experiment in A was determined as described in the Materials and Methods. The perinuclear and cytosolic zones were defined as reaching 0–2 μm and 2–5 μm, respectively, from the nuclear boundary. The distal zone was defined as any area beyond the distal limit of the cytosolic region. The distribution of mitochondria between these different zones was compared by 2‐way ANOVA; *n* ≥ 30; error bars in the graphs represent standard deviation. (*n*, number of biological replicates; ns, not significant; *P ≤ 0.05; **P ≤ 0.01; ****P ≤ 0.0001).

Since the A‐769662‐induced MMP increase could not be explained by transcriptional effects, we next assessed mitochondrial morphology that reportedly responds to AMPK activity and can impact MMP [48, 49]. Mitochondrial network analysis revealed a decrease in the summed length of branches and the number of branches in the mitochondrial network in A‐769662‐treated uninduced and Polɣ^D1135A^‐expressing cells (Fig. 7A–C). These findings of shorter and fewer branches in the mitochondrial network were observed in both U2OS and HEK cells following A‐769662 treatment and are well in line with the mitochondrial fragmentation that has been reported following AMPK activation [49]. Strikingly, both uninduced and Polɣ^D1135A^‐expressing U2OS cells treated with A‐769662 showed a smaller mitochondrial footprint as the mitochondria accumulated largely in the perinuclear region of the cell (Fig. 7D,E). Following A‐769662 treatment, the average percentage of mitochondria in the perinuclear zone of the cell increased from 15% to >55% in both uninduced and Polɣ^D1135A^‐expressing cells (from 15 to 74% in uninduced cells, and from 15% to 59% in induced cells; Fig. 7E). These findings indicate that pharmacological activation of AMPK changes mitochondrial morphology and dynamics to stimulate fragmentation and perinuclear clustering of the mitochondrial network.

### Pharmacological activation of AMPK by A‐769662 boosts MMP also in patient fibroblasts with mild mtDNA depletion

**Fig. 8:**
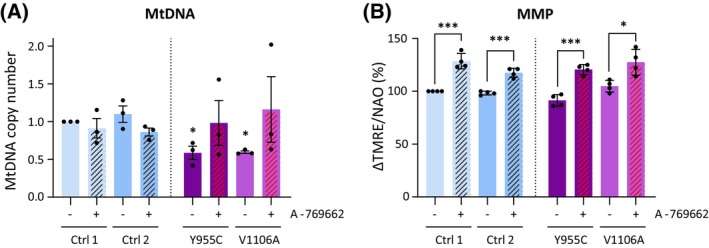
A‐769662 treatment increases MMP in patient fibroblasts. (A) Relative mtDNA copy number from human primary fibroblast cells (2 controls; 2 patient cell lines bearing the Polγ homozygous mutations Y955C and V1106A, respectively) was measured before and after treatment with A‐769662 (100 μm) for 24 h. *n* = 6. Asterisks on untreated samples indicate the *P*‐value of the comparison with the untreated Control 1 cell line; the asterisk on the A‐769662‐treated Ctrl2 sample marks the comparison with the untreated Ctrl2. (B) The mitochondrial membrane potential (MMP) of human primary fibroblast cells treated or not with A‐769662 (100 μm) for 72 h was estimated by flow cytometry as described in Fig. 1L. Relative MMP is expressed as ΔTMRE/NAO. *n* = 4. Results were analyzed by unpaired *t* test; error bars in the graphs represent standard deviation. (*n*, number of biological replicates; ns, not significant; *P ≤ 0.05; ***P ≤ 0.001).

So far, our experiments were carried out in cells expressing a dominant‐negative Polɣ mutant with highly deleterious effects on mtDNA maintenance and mitochondrial function. Given that specific AMPK activation imparted some modest but promising effects even under this extreme scenario, we next applied A‐769662 to cells with a milder and more clinically relevant level of mtDNA depletion. To this end, we made use of fibroblasts derived from patients suffering from mitochondrial myopathy, as well as sex‐ and age‐matched control cell lines. The mutant lines were homozygous either for PolɣA^V1106A^ or for the PolɣA^Y955C^ mutation that is the most common autosomal dominant mutation in *POLG* and a cause of progressive external ophthalmoplegia (PEO) [50]. Both mutant lines showed a depletion of mtDNA to approximately 60% of the levels found in control cells (Fig. 8A; 61% and 54% in Polɣ^Y955C^ and Polɣ^V1106A^ cells, respectively). Treatment with A‐769662 led to a slight increase that lacked statistical significance in patient cell lines, and no change or a decrease in the control lines (Fig. 8A). However, A‐769662‐treatment resulted in an elevation of MMP, indicating that it enhanced mitochondrial function (Fig. 8B). As was seen earlier in uninduced *vs*. induced Polɣ^D1135A^ cells (Fig. 4C), the impact of A‐769662 was observed in both control and patient cell lines, with treatment on average increasing MMP to 125% of starting values.

Taken together, the experimental findings in Figs 4, 5, 6, 7, 8 indicate that specific AMPK activation using A‐769662 has a beneficial impact on mitochondrial function as assessed by MMP both in the Flp‐In T‐REx 293 model system with severe mtDNA depletion and in mitochondrial myopathy‐associated cells (Figs 4C, 8B). The AMPK‐mediated increase in MMP following A‐769662 treatment alleviated, but did not fully eliminate, the transcriptional signature of the ISR (Fig. 6). Finally, in the Polɣ^D1135A^‐expressing cells suffering from severe mtDNA depletion and an apparent energy crisis, A‐769662 led to a partial rescue of mtDNA copy number and RC protein levels (Fig. 5A–G). The results of this study indicate that AMPK‐specific activators can assert beneficial effects in cells suffering from mitochondrial defects caused by mtDNA depletion.

## Discussion

Given its role as a master metabolic regulator tasked with ensuring energy homeostasis and adaptability to changing metabolic demands, AMPK naturally serves as a guardian of mitochondrial integrity and function. This multifaceted role involves regulation of mitochondrial biogenesis, dynamics and morphology as well as the quality control of mitochondria through mitophagy [6]. AMPK signaling also helps determine mitochondrial mobility in neuronal axons, essentially concentrating mitochondria at sites of energetic stress [51].

In the current study, we concretely show that AMPK activity is essential for maintaining MMP in healthy cells, and that mitochondria‐associated AMPK is activated early on during the progression of mitochondrial dysfunction, in line with its established role in restoring energy homeostasis (Figs 3, 4E). Here it is relevant to note that the phosphorylation level of cytosolic AMPK was not affected, and that assessment of total cellular AMPK would yield a very different result in terms of the timing of activation than that on mitochondria‐associated AMPK (AMPK activation on day 9 *vs*. day 3 in total extracts and mitochondrial fractions, respectively). Our findings suggest that the mitochondrial pool of AMPK is the first to respond to the ensuing mitochondrial dysfunction in this cell model that aims to mimic the nature of chronic and progressing mitochondrial disease. These results align well with previous findings of rapid activation of mitochondrial AMPK following more acute and extreme but perhaps less physiological mitochondrial stresses like RC inhibitors [40, 52].

Further promotion of AMPK activity using the specific small‐molecule activator A‐769662 was sufficient to recover normal MMP in Polɣ^D1135A^‐expressing cells suffering from severe mitochondrial dysfunction following mtDNA depletion (Fig. 4). Activator treatment also alleviated the transcriptional signature of the integrated stress response in these cells and led to a modest increase in mtDNA copy number and RC protein levels (Figs 5, 6). However, since A‐769662 treatment increased MMP also in uninduced control cells as well as in fibroblasts from mitochondrial myopathy patients and healthy controls where no mtDNA increase was observed (Figs 4, 8), we conclude that the stimulation of mtDNA copy number is not the general mechanism through which A‐769662‐mediated activation of AMPK stimulates MMP. A‐769662 did also not induce major transcriptional changes (in uninduced cells) or promote mitochondrial hyperfusion that in other contexts has been shown to boost MMP [48]; in contrast, activator treatment induced mitochondrial fragmentation (Figs 6, 7). Intriguingly, A‐769662‐treated cells showed clustering of the mitochondria into the perinuclear region of the cell, with fewer mitochondria found in more distal regions of the cell (Fig. 7). Perinuclear mitochondrial clustering has also been observed following stresses like depolarization or aberrant transport of mitochondria, hypoxia, and heat stress and has been reported to modulate the intensity of mito‐nuclear signaling [53, 54, 55, 56]. Notably, in our experiments, the collapse of the mitochondrial network into the perinuclear region did not correlate with MMP, as it was observed following A‐769662 addition even in uninduced cells with normal MMP, and did not occur in untreated Polɣ^D1135A^‐expressing cells with a decreased MMP (Fig. 7). Since AMPK regulates mitochondrial dynamics along the cytoskeleton [39], it is interesting to speculate that AMPK‐controlled redistribution of mitochondria to the perinuclear region could be a link in the retrograde signaling pathway triggered by mitochondrial distress and resulting in a compensatory increase in MMP. Clearly, further experimental work is needed to dissect the potential connections between subcellular redistribution of mitochondria, AMPK activity and the regulation of MMP. Moreover, given the central role of AMPK in mitochondrial metabolism, the MMP increase following A‐769662‐mediated AMPK activation is likely to be mediated by more than one mechanism. Other potential routes of action not addressed in this study include, for example, changes in substrate flux to the mitochondrial catabolic pathways, upregulation of their activity, the increased turnover of mitochondria via mitophagy and/or improved protein import as a result of increased MMP.

The observed differential effects of A‐769662 on control cells *vs*. Polɣ^D1135A^‐expressing cells with severe mtDNA depletion are intriguing. Why does AMPK activation lead to different outcomes depending on the cellular context? One possible explanation is that in mtDNA‐depleted cells, the increased AMP or ADP levels contribute to coactivation of AMPK such that a higher activity is reached than with A‐769662 alone. This hypothesis is supported by the fact that AMP and A‐769662 bind different sites of the heterotrimeric AMPK complex—while AMP binds the cystathionine‐β‐synthase (CBS) domains on the regulatory ɣ‐subunit, A‐769662 binds the allosteric drug and metabolite‐binding (ADaM) site located between the kinase domain of the catalytic ⍺‐subunit and the carbohydrate‐binding module of the β‐subunit [19]. Bultot *et al*. observed considerable coactivation of AMPK complexes derived from mouse hepatocytes or C_2_C_12_ myotubes using activators that bound to these two distinct sites [57], so we speculate that the same may be occurring in the Polɣ^D1135A^‐expressing cells treated with A‐769662 in our study. The fact that A‐769662 treatment caused no increase in mtDNA levels in the patient‐derived fibroblasts or controls—none of which are expected to suffer from a severe energy crisis comparable to that of Polɣ^D1135A^‐expressing cells—is well in line with this reasoning. Although we did not measure the adenine nucleotide pools in the patient fibroblasts, their MMP is indistinguishable from that of the control cells, suggesting that their mitochondrial ATP production and thus cellular energy status is in the normal range.

In humans, each of the three AMPK subunits is present in multiple isoforms (two isoforms each of ⍺ and β, three of ɣ), theoretically giving rise to up to 12 distinct heterotrimeric AMPK complexes. The different AMPK complexes may vary in terms of tissue‐specificity, subcellular localization and/or response to stress stimuli [6]. A shortcoming of A‐769662 in the context of targeting mitochondrial stress is that it only activates AMPK complexes containing a β1‐, but not a β2‐subunit [58], and is therefore not expected to exert an effect on AMPK complexes associated with skeletal muscle mitochondria that primarily contain β2‐subunits [40]. In contrast, compound 991 (also known as ex229) binds the same ADaM site on AMPK as A‐769662 but is 5–10 times more potent and shows no isoform specificity regarding the β‐subunit [19, 59], highlighting it as the preferential AMPK agonist for further studies.

The targeting of a central regulator like AMPK as a treatment strategy comes with its own risks and challenges. In some studies, the chronic activation of AMPK has shown undesirable side effects such as cardiac and kidney hypertrophy and Alzheimer's disease, and persistent activation of AMPK can trigger apoptosis [60, 61]. In line with those findings, cells with chronic RC deficiency have been found to downregulate AMPK signaling [62]. However, clinical trials and animal studies with other AMPK agonists did not reveal harmful side effects, indicating that activating this central regulator is feasible and safe with the right compounds [63, 64, 65, 66]. In conclusion, the positive effects of AMPK stimulation on mtDNA levels and/or mitochondrial membrane potential in cell lines with mild to severe mtDNA depletion broaden the potential applications of small‐molecule AMPK agonists and support further exploration of the effects of this group of compounds in the context of mitochondrial dysfunction.

## Materials and methods

### Cell culture

Unless otherwise indicated, cells were grown in low‐glucose DMEM medium (Gibco, Life Technologies, Paisley, UK) containing 1 g·L^−1^ glucose, 4 mm GlutaMAX™, 1 mm sodium pyruvate, 50 μg·mL^−1^ uridine, 10% heat‐inactivated fetal bovine serum (Gibco, Life Technologies, Grand Island, NY, USA) and were cultivated in a 37 °C humidity incubator at 8% CO_2_. Inducible Flp‐in™ T‐Rex™ 293 cell lines (female) (RRID: CVCL_0045) carrying one integrated copy of *myc*‐*POLG* (wt or D1135A) were generated previously [30]. The parental Flp‐In™ T‐REx™ U2OS cell line (RRID: CVCL_0042), a kind gift from Dr. Alessandro Sartori (University of Zürich), was simultaneously transfected with pOG44 (Invitrogen, Life Technologies, Carlsbad, CA, USA) and the pcDNA5/FRT/TO vector encoding a C‐terminally myc‐tagged *POLG* ^*D1135A*^ to generate a cell line carrying one integrated copy of *myc*‐*POLG* ^*D1135A*^. Expression of myc‐Polɣ was induced by the addition of 3 ng·mL^−1^ doxycycline (Sigma, St. Louis, MO, USA) to the medium for the time periods indicated in the legends. Doxycycline‐containing media was exchanged every 2–3 days. When indicated in the figures, AMPK activity was modulated by the administration of its pharmacological agonist, A‐769662 (Sigma, St. Louis, MO, USA), while control, ‘untreated’ cells received an equivalent volume of DMSO. The ρ^0^ cells used as a control were generated by 44‐day treatment of the *myc*‐*POLG* ^*D1135A*^ Flp‐In™ T‐REx™ 293 cell line with 150 ng·mL^−1^ ethidium bromide, and the lack of mtDNA confirmed by qPCR (Fig. 1B). Growth of the ρ^0^ cells was in high‐glucose (4.5 g·L^−1^) DMEM media supplemented as above. Where indicated, cells in DMEM medium were treated with 2000 μJ·cm^−2^ of UV radiation in a UV crosslinker (UVP, CL‐1000, Jena, Germany) and harvested for protein extraction 1 h after irradiation.

Human primary fibroblasts derived from two patient biopsies bearing the PolɣA mutations Y955C or V1106A, both homozygous, were cultivated in the same conditions as the Flp‐In T‐REx 293 cells. The control cells used in parallel were sex‐ and age‐matched. The research presented in this article complies with all relevant ethical regulations and the standards set by the Declaration of Helsinki. Written informed consent was obtained from all participants or their guardians. This study was approved by the Queen Square Research Ethics Committee, London, UK (09/H0716/76).

### Cell proliferation

Growth curves were generated by seeding 20 000 cells·well^−1^ in 12‐well plates. Each day, cells from three wells per condition were trypsinized, cells were resuspended in medium and counted using an automated cell counter (Countess II FL; Invitrogen, Life Technologies, Singapore). Trypan blue was used to assess cell viability and to exclude dead cells during cell counting.

### MtDNA copy number

Total DNA was isolated from cells at 80% confluency in a 6‐well plate using the NucleoSpin® Tissue DNA isolation kit (Macherey‐Nagel, Duren, Germany), according to the manufacturer's instructions. MtDNA copy number was analyzed essentially as previously described [67]. Short targets in both the mtDNA and the nuclear DNA were quantified in duplicate by quantitative real‐time PCR using 6–12 ng total DNA in a 20 μL reaction containing 0.2 μm forward and reverse primers and 10 μL of 2 × SyGreen Mix (qPCRBIO #PB20.14‐05, London, UK) in a LightCycler 96 instrument (Roche, Mannheim, Germany). Primer pairs targeted the 16S rDNA region in the mtDNA (forward GTCAACCCAACACAGGCATGCT, reverse CGTGGAGCCATTCATACAGGTCC); and a region of the single‐copy XPC gene in the nuclear genome (forward GCTGGACCATCTGCTGAACCC, reverse TCCTTCCACCCCTCACCTTATGT). All primers were confirmed to target only the single target region of the genome using the BLAST tool. Cycling conditions were as follows: 95 °C 180 s, 35 cycles of (95 °C 10 s, 57 °C 10 s, 72 °C 20 s with signal acquisition), melting curve (95 °C 5 s, 65 °C 60 s, heating to 97 °C at 0.2 °C·s^−1^ with continuous signal acquisition). C_q_ values determined by the LightCycler 96 software (Roche, Mannheim, Germany) were used to calculate the copy number of mtDNA relative to nuclear DNA using the Pfaffl method [68] and plotted with GraphPad Prism software (version 10; GraphPad Software Inc, CA, USA).

### Protein extraction and western blotting

Cells were grown to 80% confluence in 6‐well plates, resuspended in cold phosphate buffered saline (PBS) and pellets were flash‐frozen in liquid nitrogen and kept in −80 °C freezer. Total protein extracts were prepared by resuspending the cell pellets in RIPA buffer (50 mm Tris/HCl pH 8.0, 150 mm NaCl, 1% NP‐40, 0.5% Na‐deoxycholate, 0.1% SDS) containing protease and phosphatase inhibitors (100 μm AEBSF‐HCl, 4 μm aprotinin, 70 μm E‐64, 110 μm Leupeptin, 5 μm pepstatin A, 5 mm NaF, 1 mm Na_3_VO_4_). DNA was sheared by passing the samples 5 times through a 27G needle. Samples were left 30 min on ice and then centrifuged for 10 min at 17 000 ***g*** at 4 °C. Supernatants containing cell lysates were transferred to new tubes, and protein concentrations were measured using the BCA method (Pierce™ BCA Protein Assay kit, Thermo Scientific, Rockford, IL, USA). Total protein extracts (10–20 μg) were diluted in Laemmli buffer and boiled at 95 °C for 5 min. Samples were loaded onto 4–20% MiniPROTEAN® TGX™ stain‐free polyacrylamide gel (Bio‐Rad, Hercules, CA, USA) and resolved by SDS/PAGE, containing Tris‐Glycine‐SDS buffer (50 mm Tris, 250 mm glycine, 0.08% SDS). Samples used for visualization of the OXPHOS complexes using the OXPHOS antibody cocktail were loaded onto 4–12% NuPAGE™ Bis‐Tris (Thermo Fisher, Life Technologies, Carslbad, CA, USA) and 1 × MOPS was used as running buffer. Proteins were then transferred to a PVDF membrane (Amersham Hybond, Buckinghamshire, UK) in presence of Tris‐Glycine (no SDS) buffer containing 20% ethanol. The membranes were blocked with Tris‐buffered saline containing 0.1% Tween‐20 (TBS‐T) and 5% skimmed milk for 1 h at room temperature (RT) and incubated overnight at 4 °C with primary antibodies diluted in 5% milk TBS‐T. Primary antibodies used in this study are: p‐AMPKα Thr172 (1:1000; Cell Signaling #2535), AMPKα (1:500; Cell Signaling #2603), p‐ACC (1:1000; Cell Signaling #3661), ACC (1:1000; Cell Signaling #3662), p‐ATM (1:1000; Santa Cruz sc‐47 739), ATM (1:1000; Santa Cruz sc‐377 293), OXPHOS cocktail (1:2000; Invitrogen 45‐8199), myc‐tag (1:1000; Invitrogen MA1‐21316), GAPDH (1:10 000; Invitrogen MA5‐15738), VDAC/Porin (1:5000; Abcam ab14734), COX2 (1:10 000; Invitrogen A6404), TFAM (1:10 000; Abcam ab176558), and β‐actin (1:15 000; Sigma A5441). Secondary antibodies (anti‐mouse (1:10 000–20 000; Pierce #31430) and anti‐rabbit (1:20 000; Themo Scientific #31460)) used were HRP‐linked diluted in 5% milk TBS‐T, followed by 1‐h incubation at RT with the membrane. After extensive wash with TBS‐T, the blots were developed by chemiluminescence (ECL Bright or ECL SuperBright, Agrisera, Sweden) and images were taken using ChemiDoc Imaging System (Bio‐Rad, Hercules, CA, USA). Image analysis and quantifications were done using imagej software (version 1.54g).

### Cell fractionation

To determine the subcellular localization of (p)AMPK, two 10 cm plates per condition were grown to 80% confluence, harvested, washed twice with cold PBS, and then resuspended and incubated in 0.1 × hypotonic buffer (4 mm Tris/HCl pH 7.8, 2.5 mm NaCl, 0.5 mm MgCl_2_ containing protease and phosphatase inhibitors) for 10 min at 4 °C. Cells were homogenized in a 1 mL glass/glass Wheaton tight‐fitting dounce (Active Motif, Waterloo, Belgium) until ca 90% of cells were disrupted, and samples made isotonic by addition of 1/10 vol of 10 × homogenization buffer. Nuclear, cytosolic, and crude mitochondrial fractions were obtained after differential centrifugation. Briefly, nuclear fractions were pelleted by centrifugation at 1200 ***g*** for 3 min, and the supernatant containing cytosolic and mitochondrial fractions was transferred to new tubes. The centrifugation was repeated twice to remove any remaining nuclear material. From the resulting supernatant, mitochondria were pelleted by centrifugation at 17 000 ***g*** for 10 min and the supernatant was collected as the cytosolic fraction. Proteins from the different fractions were extracted and quantified as described above.

Where noted, the crude mitochondrial fraction was further subjected to density gradient centrifugation to separate mitochondria from lysosomes as previously described [69] with some modifications. Cells were grown on five 15‐cm plates to 90% confluency before being harvested and subjected to cell fractionation as described above to obtain the crude mitochondrial fraction. HistoDenz (Htz; Sigma, St. Louis, MO, USA) was used as a more stable and less toxic alternative to metrizamide [70]. Briefly, the crude mitochondrial pellet was carefully resuspended in 0.25 m sucrose pH 7.2 and loaded on the top of a gradient (from bottom to top: 35% Htz, 17% Htz, 6% Percoll in a 13.2 mL ultra‐clear tube (344 059; Beckman)). The ultracentrifugation was run at 70 000 ***g*** for 35 min at 4 °C in a SW 41 Ti rotor, and the light mitochondrial fraction that contained enriched mitochondria and lysosome was collected from the 17% Htz/6% Percoll interface. The two compartments were separated by mixing the fraction with 80% Htz at the bottom of an Ultra‐Clear tube and layering a Histodenz gradient (from bottom to top: 17% Htz, 5% Htz, 0.25 M sucrose). After ultracentrifugation at 70 000 ***g*** for 35 min at 4 °C, the lysosomal and mitochondrial fractions were collected from the 5%/17% Htz interface and 17%/35% Htz interface, respectively. To pellet the lysosomes/mitochondria, the collected fractions were diluted with at least five volumes of ice‐cold PBS and centrifuged at 20 000 ***g*** for 30 min at 4 °C. The purity of lysosomal fractions was analyzed by western blot using antibodies against lysosomal marker LAMP‐1 (1:5000, Cell Signaling #15665); other antibodies were as mentioned under ‘Protein extraction and Western blotting’.

### Blue native PAGE (BN‐PAGE) and CIV in‐gel activity

To assess the integrity and function of the mitochondrial complexes and supercomplexes, mitochondria were isolated from harvested cells, solubilized, and resolved in blue native PAGE to further perform CIV in‐gel activity determine and protein levels by immunoblotting according to a protocol adapted from [71]. Briefly, cells from two 150 mm plates per condition were harvested at 90% confluence, suspended in IBc buffer (10 mm Tris‐MOPS, 1 mm EGTA, 0.2 m sucrose) containing protease and phosphatase inhibitors, and homogenized in a 15 mL glass/glass Wheaton tight‐fitting dounce (Active Motif, Waterloo, Belgium) until they were 55–65% disrupted (positive for Trypan blue staining according to automated cell counting (Countess II FL; Invitrogen)). Mitochondrial pellets were obtained by differential centrifugation, resuspended in IBc buffer and the proteins quantified using the BCA method. Aliquots of mitochondrial fraction were flash‐frozen in liquid nitrogen and stored at −80 °C for up to 2 weeks. On the day of the assay, mitochondrial pellets were thawed on ice and solubilized in NativePAGE™ buffer (Invitrogen) containing 4% digitonin and protease inhibitors. After 1.5‐h incubation with solubilization buffer, the samples were centrifuged at 16 000 ***g*** for 20 min and the supernatant containing solubilized mitochondria was mixed with NativePAGE™ G‐250 sample additive (Invitrogen). Mitochondrial samples (100 μg for in‐gel activity; 25 μg for WB) were loaded onto NativePAGE™ 3–12% Bis‐Tris gel (Invitrogen) and resolved by BN‐PAGE containing NativePAGE™ Running buffer (Invitrogen) and NativePAGE™ cathode buffer additive (Invitrogen). NativeMark™ Unstained Protein Standard (Invitrogen) was used for molecular weight estimation of protein in native gel electrophoresis. To assess the activity of CIV, the gel was incubated overnight with substrate solution (50 mm Na‐phosphate buffer pH7.4, 0.5 g·L^−1^ 3,3′‐diaminobenzidine tetrahydrochloride, 1.0 g·L^−1^ bovine cytochrome *c*, 2 mg·L^−1^ catalase, 75 g·L^−1^ sucrose) until the brown signal appeared on the gel. Images of the gel were recorded using a high‐quality imager (Epson Perfection V700 Photo).

### Mitochondrial membrane potential (MMP)

Cells were grown to 70–80% confluence in 6‐well plates. For each condition, one well was used for tetramethylrhodamine ethyl ester (TMRE) staining and another for 10‐N‐nonyl acridine orange (NAO) staining. To assess MMP, adherent cells were incubated with medium containing 200 nm TMRE for 30 min at 37 °C. After incubation, cells were trypsinized and resuspended in PBS containing 1% BSA, and TMRE fluorescence was determined by flow cytometry (BD Accuri™ C6 Plus; BD Biosciences). TMRE fluorescence was also measured in cells further treated with the mitochondrial uncoupler BAM‐15 (60 μm) for 30 min at 37 °C to exclude nonmitochondrial TMRE fluorescence. Mitochondrial mass was estimated by the fluorescence of cells stained with 10 nm NAO for 30 min at 37 °C. The MMP was calculated by subtracting the TMRE fluorescence of BAM‐15 uncoupled cells from the respective coupled ones (ΔTMRE = TMRE_coupled_ – TMRE_uncoupled_) and the result was normalized by the NAO fluorescence (ΔTMRE/NAO).

### AMPK silencing

AMPK silencing was achieved via cell transfection with AMPKα1/α2 siRNA (#45312; Santa Cruz), and scrambled siRNA (#4390844; Thermo Fisher) was used as negative control. Shortly, 50% confluent wells (6‐well plate) were transfected with 100 pmol siRNA and Lipofectamine® RNAiMAX (Invitrogen) prepared in OptiMEM® (Gibco) medium, according to manufacturer. After 24 h transfection, the medium was replaced with fresh medium containing either DMSO or 100 μm A‐769662, and cells were harvested 48 h later for MMP measurement and WB analysis.

### Nucleotide pool measurement

Nucleotide (dNTP and NTP) pools were measured in a single run using a recently developed isocratic reverse phase HPLC‐based technique [72], with minor modifications for the analysis of the relative levels of AMP, ADP and ATP [73, 74]. Briefly, cells were cultivated in 10 cm plates to 50–75% confluence, washed with ice‐cold PBS, and scraped off the plates in the presence of 0.5 mL ice‐cold 80% (v/v) methanol in water. Cells were centrifuged for 1 min at 17 000 ***g*** in a cooled centrifuge, and the supernatants containing the free nucleotides were collected in new tubes and stored at −80 °C. The cell extracts were purified by solid phase extraction (SPE) using Oasis WAX 3 cc cartridges (Waters Corporation, Milford, MA, USA) in a similar manner as described before for the measurement of NTPs, dNTPs, and ADP [72], but with some minor modifications in the SPE step in experiments where also AMP was measured [74]. The SPE‐purified samples were subsequently analyzed by HPLC run at 1 mL·min^−1^ using a 150 mm × 4.6 mm SunShell C18‐WP HPLC column from ChromaNik Technologies Inc (Osaka, Japan). The aqueous mobile phase contained 5.8% (v/v) acetonitrile, 0.7 g·L^−1^ tetrabutylammonium bromide as ion pairing agent and varying concentrations of potassium phosphate at pH 5.6. The analysis of NTPs and dNTPs was performed with the isocratic Fast Protocol (Ranjbarian *et al*., 2022), whereas the analysis of AMP, ADP, and ATP was performed with a phosphate gradient [73].

### Cell cycle

Cells were grown to 70–80% confluence in 6‐well plates, harvested and fixed with ice‐cold 70% ethanol, and kept in freezer −20 °C up to 1 week. For DNA staining, cells were washed with PBS and resuspended in staining solution containing 0.02 mg·mL^−1^ propidium iodide, 1 mm EDTA, 0.1% Triton X‐100, and 0.2 mg·mL^−1^ RNAse in PBS. Cells were incubated with staining solution for 30 min at RT. Cell cycle was assessed by flow cytometry (BD Accuri™ C6 Plus; BD Biosciences), and data were analyzed using FlowJo™ BD software.

### Cell death assay

Levels of cell death were measured in cells expressing Polɣ^WT^ and Polɣ^D1135A^ for 6 days using the FITC/Annexin V Dead Cell Apoptosis kit (Molecular Probes; Invitrogen) according to the manufacturer's instructions. Uninduced Polɣ^WT^ cells treated with 10 μm of camptothecin for 16 h were used as a positive control for cell death. Cells were harvested, pelleted by centrifugation, washed once with 1 × PBS, and resuspended in PBS to a concentration of 10^6^ cells·mL^−1^. For each measurement, 10^5^ cells were combined with 5 μL FITC Annexin V solution and 1 μL of 100 μg·mL^−1^ propidium iodide solution. The samples were incubated at RT, protected from light, for 15 min. After incubation, 400 μL of annexin binding buffer was added to the mixture, gently mixed, and kept on ice. Samples were measured immediately after the addition of the buffer on a BD Accuri C6 Plus (BD Biosciences) flow cytometer. Unstained and uninduced cells were used as a negative control.

### RNA‐seq analysis

Uninduced and 4‐day induced Polɣ^D1135A^ cells untreated or treated with 100 μm A‐769662 for 72 h were grown to 80–90% confluency in 6‐well plates, harvested, and flash‐frozen. Total RNA was extracted from cell pellets using Qiagen RNeasy Plus kit (Qiagen) according to manufacturer's instructions. Initial sample quality control was performed using Qubit and agarose gel electrophoresis. RNA libraries were prepared by Azenta Life Sciences using Illumina's PolyA selection method, followed by sequencing on the Illumina NovaSeq platform with a 2 × 150 bp configuration, achieving a sequencing depth of approximately 20–30 million paired end reads per sample. Data files were assessed using FastQC, and read alignment was performed using STAR2.5.2b. On the WSL2 platform, featureCounts for Linux was utilized for quantification and generation of counts files. Raw count data were prefiltered to remove low‐count genes, retaining those with at least 10 counts across all samples. Differential expression analysis was performed on normalized counts using DESeq2 in R 4.2.3, and log_2_ fold change (log_2_FC) values for each condition were calculated using the apeglm shrinkage method [75]. Comparisons were made across all conditions, gene names retrieved using the biomaRt package, and a list of significantly differentially expressed genes was generated by filtering for genes with an adjusted *P*‐value (*P*adj) of <0.05 (Table S1). Genes with a considerable change in expression (log_2_FC > |1|) are listed in Table S2. Finally, a list of ‘rescued’ genes was generated containing genes from Table S1 that were differentially expressed in the comparison Uninduced *vs*. D1135A, but not in the comparison Uninduced+A‐76 *vs*. D1135A + A‐76 (Table S3). Heatmaps were generated to visualize the expression patterns, with coloring relating to the log_2_FC relative to the Uninduced condition.

STRING (Search Tool for the Retrieval of Interacting Genes/Proteins) database (v12.0, 2023) analysis was conducted using RStudio 2023.12.1 Build 402 [76]. Initially, protein identifiers corresponding to the list of rescued genes were mapped across multiple databases to standardize the data and ensure comprehensive information on specific identifiers. Gene Ontology (GO) enrichment analysis was performed to identify biological processes, molecular functions, and cellular components in the retrieved gene list. The results were then analyzed for protein–protein interactions using the STRING database API to uncover known functional connections and enrichments. Network data were filtered to highlight gene‐specific connections, emphasizing common nodes between two or more networks. Domain and KEGG (Kyoto Encyclopedia of Genes and Genomes, v112.0, 2024) [77] enrichment analyses were performed to identify pathways associated with specific networks.

### Immunofluorescence microscopy and image analysis

Inducible Flp‐In™ T‐REx™ U2OS Polɣ^D1135A^ cells were grown for 6 days in the absence or presence of 3 ng·mL^−1^ doxycycline, with medium refreshed every 48 h. A‐769662 treatment was for 48 h, with 100 μm A‐769662 added for the last 2 days of induction. Cells were then fixed with 3.3% paraformaldehyde (PFA) for 20 min in precoated Ibidi μ‐Slide 8 well chambers (Ibidi: 80826). Following fixation, cells were washed three times with PBS and permeabilized with 0.1% Triton X‐100 for 10 min. Cells were then washed three more times with PBS and incubated in blocking solution (PBS containing 5% BSA) at room temperature for 1 h. To stain the mitochondria, cells were incubated overnight at 4 °C with TOM20 antibody (1:500; Santa Cruz: sc‐17 764), followed by incubation with Alexa Fluor 488‐conjugated secondary antibody (1:300; Thermo Fisher Scientific: A‐11001) for 1 h at room temperature and protected from light. Both antibodies were diluted in blocking solution. DNA was stained using 1 μg·mL^−1^ 4′,6‐diamidino‐2‐phenylindole (DAPI) in PBS for 10 min. Between each incubation step, cells were washed three times with PBS. Images were captured using a Leica SP8 confocal microscope with a 63x oil immersion objective.

Mitochondrial network analysis was performed using fiji software version 2.4.0/1.54 with the MiNA (Mitochondrial Network Analysis) plugin. Treated and induced cells were compared to uninduced controls for each replicate, and three biological replicates were analyzed. The area occupied by mitochondria was quantified by measuring the space covered by the fluorescence signal in the mitochondrial channel using imagej and comparing the value to the total area of the cell.

To assess the perinuclear clustering of mitochondria, immunofluorescence images were analyzed using the Radial Profile plugin in fiji software version 2.4.0/1.54. The images were split into each respective fluorescence channel, and a circular region of interest (ROI) was established to cover the entire cell. The same ROI was applied to all channels, and a radial profile was generated based on the signal intensity of each pixel in the DAPI and mitochondrial marker channels. Since the size of the nucleus can vary from cell to cell, the location of the nuclear boundary in each cell was determined individually and was set at the point relative to the center of the cell where the DAPI signal intensity dropped to below 30% of its peak value. The perinuclear region was defined as a circular zone with a radius that extended 2 μm beyond this nuclear boundary, and the cytosolic region was defined to be between 2 and 5 μm from the nuclear boundary. The distal zone was defined as any area beyond the distal limit of the cytosolic region. The intensity of the mitochondrial signal in each zone was quantified using the radii calculated for each zone in each particular cell and expressed as a percentage of the total mitochondrial signal in that cell. At least 30 cells were individually measured for each condition.

### Graphs and statistical analysis

All graphs and statistical analyses were generated with GraphPad Prism software (version 10; GraphPad Software Inc, CA). For experiments comparing uninduced and induced Flp‐In T‐REx 293 cells across multiple timepoints, data of all induced samples were normalized to the induced sample at day 0 to eliminate technical variation arising from different doxycycline preparations. To clearly indicate this, a vertical dotted line is used to divide the graphs into two halves, where data sets in each half are normalized to their respective 0‐day sample. Results are expressed as mean ± standard deviation, and statistical differences (unpaired *t* test) indicated by asterisks (*P ≤ 0.05, **P ≤ 0.01, ***P ≤ 0.001, ****P ≤ 0.0001). The number of biological replicates (*n*) is indicated in the figure legends.

## Conflict of interest

The authors declare no conflict of interest.

## Author contributions

GC—conceptualization, investigation, methodology, formal analysis, data curation, validation, visualization, project administration; BR, TVHN, JMEF, FR, ICM, WMRRW, CMG, and NC—investigation, methodology, formal analysis, validation; MF and RDSP—conceptualization, resources; MD—investigation, validation; SW—methodology, supervision, funding acquisition; AH—methodology, formal analysis, supervision; PHW—conceptualization, formal analysis, data curation, funding acquisition, project administration, supervision, writing‐original draft. All authors reviewed and edited the final version.

### Peer review

The peer review history for this article is available at https://www.webofscience.com/api/gateway/wos/peer‐review/10.1111/febs.70006.

## Supporting information


**Table S1.** Gene comparisons. List of significantly differentially‐expressed genes (DEGs) from the RNA‐seq analysis.

**Table S2.** Gene comparisons filtered on log_2_FC. List of significant DEGs from the RNA‐seq analysis, filtered for |log_2_FC| ≥1.

**Table S3.** Rescued genes. List of genes that were differentially‐expressed in induced Polɣ^D1135A^ cells but not in induced Polɣ^D1135A^ cells treated with A‐769662.

## Data Availability

The RNA‐seq data that support the findings of this study are openly available in NCBI's Gene Expression Omnibus and are accessible through https://www.ncbi.nlm.nih.gov/geo/query/acc.cgi?acc=GSE282399 with the GEO Series accession number GSE282399.
